# Genetic Characterization of Primordial Germ Cells in Spotted Sea Bass (*Lateolabrax maculatus*)

**DOI:** 10.3390/genes16091012

**Published:** 2025-08-27

**Authors:** Jieyun Guo, Lulu Yan, Chao Zhao, Bo Zhang, Bo Zhang, Lihua Qiu

**Affiliations:** State Key Laboratory of Mariculture Biobreeding and Sustainable Goods (BRESG), Guangdong Provincial Key Laboratory of Fishery Ecology and Environment, Key Laboratory of South China Sea Fishery Resources Exploitation & Utilization, Ministry of Agriculture and Rural Affairs, South China Sea Fisheries Research Institute, Chinese Academy of Fishery Sciences, Guangzhou 510300, China; 22jjguo@stu.edu.cn (J.G.);

**Keywords:** PGC, vasa, dead end, microinjection, 3′-UTR, spotted sea bass

## Abstract

Background: Primordial germ cells (PGC) are the progenitor cells of sperm and eggs during the embryonic stage. The maternal gene vasa has been widely studied for its role in PGC origin, and other genes like *dead end* (dnd) have also been identified. Objectives: Spotted sea bass is an important economic marine fish, and the study of its germ cell characteristics provides important basic data for future population breeding and protection. Methods: In this study, we cloned the full-length sequences of *Lmvasa* (2384 bp, encoding 1905 aa) and *Lmdnd* (1523 bp, encoding 386 aa) using RACE. Temporal and spatial expression patterns of Lmvasa and Lmdnd in embryos and gonads were analyzed by PCR, immunohistochemistry, and in situ hybridization. We also used microinjections of chimeric RNA containing GFP and *Lmvasa* 3′ UTR to visualize PGCs. Results: Our results showed that *Lmvasa* and *Lmdnd* are expressed primarily in early embryonic development (pre-blastula stage) and were expressed only in the gonads. Immunohistochemistry revealed abundant expression of Lmvasa and Lmdnd proteins in spermatogonia, weak expression in spermatocytes, and no expression in spermatozoa. In ovaries, both genes were expressed throughout oogenesis. Furthermore, PGCs in spotted sea bass belonged to an early localization pattern. Microinjection experiments demonstrated that *Lmvasa* 3′ UTR effectively labeled PGCs in embryos of spotted sea bass, zebrafish, and medaka. Conclusions: These findings may contribute to understanding PGC development in spotted sea bass and other Percidae.

## 1. Introduction

As sexually reproducing organisms, fish are composed of somatic and germ cells. The pre-sexual germ cells (i.e., primordial germ cells (PGCs)) are specialized from cells that acquire germ plasm components during early embryonic development [1]. PGCs enter the primordial genital ridges through various pathways, form the primordial gonads, and differentiate into mature germ cells [2]. These cells pass on genetic information from generation to generation, which allows species to develop and evolve.

Initial studies found that the germ cells of anuran amphibians contain an electron-dense structure rich in mitochondria, which was named germ plasm [3]. Such electron-dense bodies are found in PGCs and also in germ cells at different stages of development. In teleosts, studies of removed [4,5] and transplanted [6] germ plasm revealed that it is essential for the identification of PGCs and the developmental process. Fruit flies (*Drosophila melanogaste*) contain a similar structure, and this germ cell is a precursor of the polar cell [7]. The pole plasm has the potential to induce germ cell formation. In addition, the maternal effector vasa is required for the normal formation of the pole cells [8]. Vasa was first discovered in Drosophila, and this polar component is involved in the regulation of germ cell translation and plays an important role in polar formation and germ cell development. Vasa belongs to the DEAD family, and its members encode ATP-dependent RNA helicases [9,10,11]. Vasa is specifically expressed in the germ cell of many organisms, and this expression pattern is highly conserved [12].

In teleosts, vasa has been used as a whole mount in situ hybridization (WISH) probe to investigate two classical modes of origin of PGCs. WISH showed that early localization is present in zebrafish (*Danio rerio*), and that vasa mRNAs are concentrated in the cleavage furrow in the early stage of cleavage [13]. In contrast, medaka (*Oryzias. latipes*) has lost the function of early localization; vasa mRNAs are distributed in various blastomeres in the early embryonic period, and they do not accumulate in certain cells until late gastrulation [14]. Germ cells in the gonads can also be identified by sectioning with vasa anti-sense probes or immunohistochemistry (IHC) of anti-vasa antibodies, especially for early germ cells such as spermatocytes and oocytes [15,16,17]. Stable germ cell labeling has also been achieved by generating transgenic animals utilizing vasa regulatory sequences, which include both the promoter and the 3′ untranslated region (UTR). For instance, transgenic zebrafish driven by the vasa promoter were able to specifically express GFP within the germ cell lineage [18]. In certain commercial fish species, such as rainbow trout (*Oncorhynchus mykiss*) and turbot (*Scophthalmus maximus*), transient labeling of PGCs with fluorescent proteins (GFP or mCherry) was accomplished in vivo through the microinjection of synthetic chimeric mRNA containing these fluorescent proteins into zygotes [19,20,21].

Dead end (Dnd) is a maternal RNA that is specific to vertebrate germ plasm, and it has been identified in zebrafish as an RNA-binding protein uniquely expressed in germ cells [22]. In zebrafish [23], *Xenopus* [24], and mouse [25], loss of *dnd* function leads to abnormal migration, apoptosis, or deletion of PGCs. Human adolescent testicular germ cell tumors are also associated with *dnd* variants, suggesting a conserved role for *dnd* in vertebrate germline development [26]. However, *dnd* mRNA expression exhibits sexual dimorphism in adult gonads across species. For example, it was detected only in mouse testis [27], whereas in *Xenopus* [28] and the catfish *(Pseudopimelodus mangurus*) [29], *dnd* mRNA expression was restricted to the ovary. Dnd has also been found in the sexual germ cells of medaka [30], zebrafish [23], spinyhead croaker (*Collichthys lucidus*) [31], and starry flounder (*Platichthys stellatus*) [32].

The spotted sea bass (*L. maculatus*) is an economically significant species in the mariculture industry in China, where it is widely cultivated due to its delicious taste and high nutritional value [33,34]. However, the relatively long sexual maturation cycle (approximately 3 years) coupled with the strict requirements for ovarian maturation limit the breeding efficiency and egg yield of this species. Due to habitat degradation, overfishing, and other reasons, the germ plasm quality of spotted sea bass has also degraded. Moreover, the introgression of breeding offspring and adult fish that have escaped from culture ponds or cages has negatively impacted the biodiversity of wild populations of spotted sea bass [35]. However, the development of germ cell technology has made it possible to transplant germline stem cells into fish to shorten the breeding cycle and improve breeding efficiency.

Previous studies have identified PGC through the expression of *Lcvasa* in adult gonadal tissue in Asian bass (*Lates calcarifer*) [36]. Recently, single-cell RNA sequencing has also validated *Lcvasa* and other germline-specific markers in adult ovaries [37]. However, studies on PGC migration pathways during early embryogenesis in Perciformes have not been reported, with a specific lack of studies on the migration pathways of Percidae. In this study, we aimed to identify suitable PGC marker genes in spotted bass and characterize PGC distribution during embryogenesis using WISH and in vivo PGC labeling techniques.

## 2. Materials and Methods

### 2.1. Fish and Sample Collection

Spotted sea bass used in this study were 2-year-olds (body length: 58 ± 2 cm, weight: 2 kg, 3 male and 3 female) sourced from ChangFeng Aquatic Sci-Tech Co., Ltd. (Zhuhai, Guangdong Province, China). Fertilized eggs were obtained by natural spawning and cultured at 18 °C ± 1 °C in fresh seawater at Hongxinrong Aquaculture Hatchery (Zhangpu, Fujian Province, China).

After anesthetizing the spotted sea bass, tissue samples (gills, liver, spleen, kidneys, brain, intestines, muscles, ovaries, and testes) were collected and stored in liquid nitrogen (*n* = 3). After laying eggs, the fertilized eggs were quickly and gently collected with a net. Embryo development was first observed under a stereo microscope (M165 FC, Leica, Wetzlar, Germany). The embryos of the same period were collected with a 5 mL straw and washed with phosphate-buffered saline (PBS). The liquid was aspirated, then transferred to a 2 mL cryopreservation tube and immediately put into liquid nitrogen (*n* = 3, embryos in each tube ≥ 20).

For IHC and WISH analyses, gonad and embryos were washed in PBS and placed in a fixation solution overnight at 4 °C. Gonad tissues and embryos were dehydrated using PBS-diluted 50% methanol solution for 2 h at room temperature, transferred to 100% methanol solution for 2 h, and finally placed in new 100% methanol solution for storage at −20 °C until used for analysis (*n* = 3, embryos in each tube ≥ 30).

The marina medaka (*Oryzias melastigma*) was purchased from Shanghai Feixi Biotechnology Co., Ltd. (Shanghai, China), and the AB zebrafish came from the Wuhan Institute of Hydrobiology (Wuhan, China). Medaka were raised in a circulation system of 30‰ seawater, and zebrafish were raised in a freshwater circulation system. They were raised at 28 °C, and the light period was 14 h in light/10 h in dark. Embryo Collection: female and male fish were reared separately the night before and mixed at a ratio of 2:1 (female:male) the next morning. The fertilized eggs were collected immediately after the female laid the eggs.

### 2.2. Total RNA Isolation and cDNA Synthesis

Spotted sea bass tissues and embryos total RNA were extracted with Trizol reagent. Reverse transcription of first-strand cDNA was performed using FastKing gDNA Dispelling RT SuperMix kit (Tiangen, Beijing, China). We amplified the open reading frame (ORF) of *Lmvasa* and *Lmdnd* from the expression sequence tags (ESTs) of the ovaries of spotted bass in this research group. To obtain full-length cDNA sequences for *vasa* and *dnd*, we designed fragments amplified from the expression sequence tags of the of the spotted sea bass in this topic, and these sequences were uploaded to the NCBI database. *Lmvasa* and *Lmdnd* full-length cDNA sequences were amplified and sequenced, then aligned using the SMARTer RACE 5′/3′ Kit (Clontech, San Jose, CA, USA). Table 1 lists all primers used in this study. During this period, the sequence was uploaded to the NCBI database, and the accession number was obtained: *vasa* (GenBank login: PV982366) and *dnd* (GenBank login: PV982367).

### 2.3. Phylogenetic Analysis

The amino acid sequences and similarity analyses of Lmvasa and Lmdnd were conducted using BLASTn and BLASTp from the NCBI databases. Jalview 2.11 and Clustal Omega 1.2.4 software were used for sequence alignment. Phylogenetic analysis was carried out using MEGA v5.2 software according to the previous method [38,39].

### 2.4. RT-qPCR and RT-PCR

RT-qPCR was performed on a LightCycler^®^ 480 II PCR (Roche, Basel, Switzerland) according to the Taq Pro Universal SYBR qPCR Master Mix Kit (Vazyme, Nanjing, China). RT-PCR was performed according to the 2× Rapid Taq Master Mix (Vazyme, Nanjing, China). *β-actin* was used as a standardized control [16,40]. For RT-PCR, *β-actin* was amplified with 25 cycles, and *Lmvasa* and *Lmdnd* were amplified with 30 cycles. Relative gene-expression levels were calculated using the 2^−ΔΔCt^ method. The primer sequences are shown in Table 1.

### 2.5. WISH and Histology

Sense and antisense *Lmvasa* and *Lmdnd* probes were synthesized from the pGEM-T easy vector containing a 1017 bp *Lmvasa* fragment and a 1155 bp *Lmdnd* fragment following a previously published method (Table 1) [41]. The resulting plasmids were linearized by Sac II and Nco I monodigestion, respectively. According to the digoxin RNA labeling kits (SP6/T7; Roche, Mannheim, Germany), purified *Lmvasa* and *Lmdnd* probes were obtained, dissolved in RNase-free water, and stored in aliquots at −80 °C. The probe concentration for *Lmvasa* and *Lmdnd* was 2 ng/μL.

For histology, the gonads, fixed as described in Section 2.2, were sent to Wuhan Servicebio Technology (China) for embedding, section treatment (4 μm), IHC, and hematoxylin-eosin (H&E) staining (*n* = 3). All sliced images were observed and acquired under a Leica (DM2500, Leica) with 20.0× and 40.0× lenses. Each slice randomly selected 10 areas for observation, and corresponding typical field of view pictures were provided. For the WISH assay, dehydrated embryos (*n* ≥ 20) from different periods were removed from storage at −20 °C and gradually diluted with methanol and 0.1% Tween 20 (PBST) at room temperature. Procedures such as embryo permeability treatment, fixation, probe hybridization, washing, chromogenic and embedding were performed according to the methods of Narayanan et al. [41] and Kimmel et al. [42]. Finally, photographs were taken using the stereomicroscope.

### 2.6. Expression of Recombinant Lmvasa Protein and Preparation of Its Antibody

Lmvasa protein was obtained by inducing the expression of the fusion protein (Lmvasa-His) following a previously established protocol [17]. Briefly, the *vasa* coding region was amplified and inserted into pET28a digested with EcoR I and Xho I using the In-fusion^®^ HD Cloning Kit (Takara, Tokyo, Japan) (Table 1). Vasa-PET28a was recombinantly expressed in *Escherichia coli* (*E. coli*) cells and purified in the HyPur T Ni-TED 6FF (His-Tag) PrePacked Gravity Column Kit (Sangon, Shanghai, China), then detected by SDS-PAGE. The laboratory-purified vasa protein was sent to Hangzhou Huaan Biotechnology (Hangzhou, China) to prepare vasa rabbit polyclonal antibodies (pAbs).

### 2.7. Western Blotting Analysis

Ovarian tissue was incubated on ice for 20 min in protein lysate containing a final concentration of 1 mM benzsulfonyl fluoride (Beyotime, Nantong, China) and transferred to SDS-PAGE. Western blot analysis of tissue lysates was basically completed as previously described [36].

The primary antibodies used to detect vasa were rabbit pAb prepared by Hangzhou Huaan Biotechnology and β-actin (HRP-conjugated) rabbit mAb (49381) purchased from Signalway Antibody LLC (Greenbelt, MD, USA).

### 2.8. Preparation of Chimeric mRNAs and Microinjection

PCS^2+^-EGFP plasmid was used as a template for RNA synthesis in vitro. GFP-*Lmvasa* 3′-UTR was constructed from previous descriptions [43]. In brief, the 3′-UTR regions were amplified and inserted into PCS^2+^-EGFP digested with Xho I-Xba I, and the In-Fusion ^®^ HD Cloning Kit was used (Table 1). The resultant plasmid was linearized by BamH I digestion for in vitro transcription using the mMESSAGE mMACHINE^®^ SP6 transcription kit (Invitrogen, Waltham, MA, USA) and stored at −80 °C until use.

As previously described [18,31], mRNA (300 ng/μL) of spotted sea bass, zebrafish, or medaka embryos was prepared. The embryos were placed in a hole on the surface of the agar, and then RNA microinjection solution was microinjected at the 1–4 cell stage for PGCs visualization. In spotted sea bass and zebrafish, each experimental group ≥500 embryos were injected (*n* = 5). In medaka, each experimental group ≥ 150 embryos were injected (*n* = 5). The control group was not injected with embryos, and there were the same number of control and experimental groups.

### 2.9. Microscopy

Observe and photograph embryos using Leica DMi8 inverted microscope DMi8 and stereomicroscope (Leica, Wetzlar, Germany).

### 2.10. Statistical Analysis

Data was presented as mean ± SEM. All statistical data analyses were performed using GraphPad Prism 10.1 software. Significant differences were assessed by one-way ANOVA and Tukey’s multiple comparison test. When *p* < 0.05, the difference was considered significant.

## 3. Results

### 3.1. Cloning and Characterization of Lmvasa and Lmdnd

Lmvasa fragments of 1905 bp were obtained from the ovaries of spotted bass using specific primers. The *Lmvasa* cDNA was obtained using 3′ and 5′ RACE techniques has a total length of 2384 bp and consists of 152 bp 5′-UTR, 635 bp ORF, encoding 1905 aa, and 327 bp 3′-UTR (Figure S1).

Similarly, the *Lmdnd* ORF was amplified using specific primers to 1158 bp. The total length of the *Lmdnd* amplified by RACE technology was 1523 bp and consists of 84-bp 5′-UTR, 1158 bp ORF encoding 386 aa, and 281 bp 3′-UTR (Figure S2).

**Figure 1:**
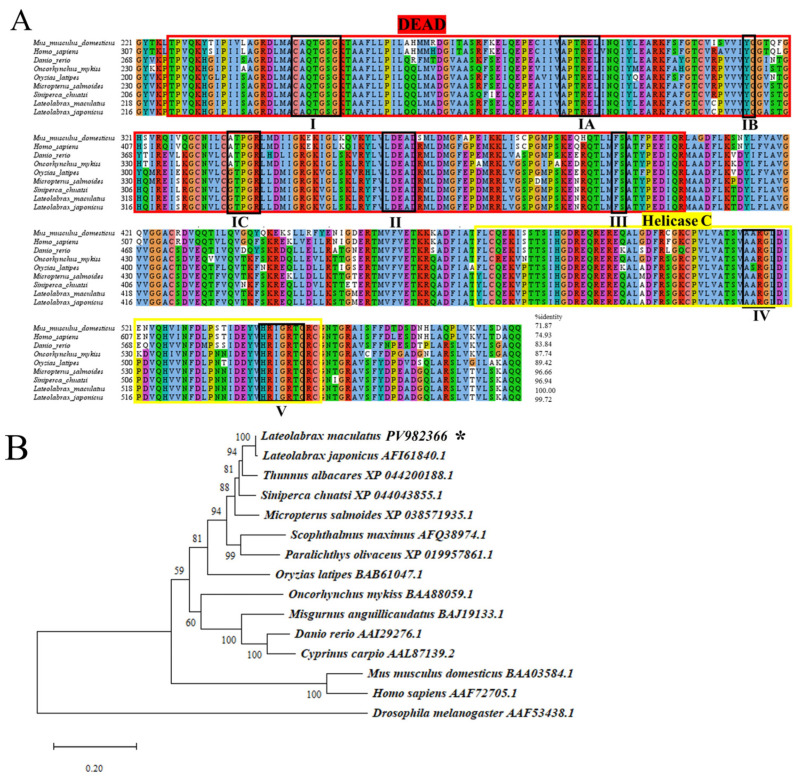
Multiple alignment and phylogenetic tree of vasa aa of spotted sea bass. (**A**) Conserved motifs are shown in the black block. The DEAD-box sequence is indicated in bright red, whereas Helicase C is in yellow. The eight domains within these superfamilies [I domain (AQTGSGKT), IA domain (PTREL), IB domain (GG), IC domain (TPGRL), II domain (DEAD), III domain (SAT), IV domain (ARGLD), and V domain (GRTGR)] are outlined with black rectangles. (**B**) Phylogenetic tree generated using the neighbor-joining method based on vasa. Other accession numbers are indicated. * represents the species that are studied.

Multiple sequence alignment revealed that Lmvasa shared high identity (71.87–99.72%) with *vasa* sequences from other fish (Figure 1A). The deduced spotted sea bass vasa protein possessed eight consensus motifs characteristic of the DEAD-box protein family, and the N-terminal region was rich in glycine (G) and arginine (R) residues-RGG and RG motifs (Figure S1 and Figure 1A).

**Figure 2:**
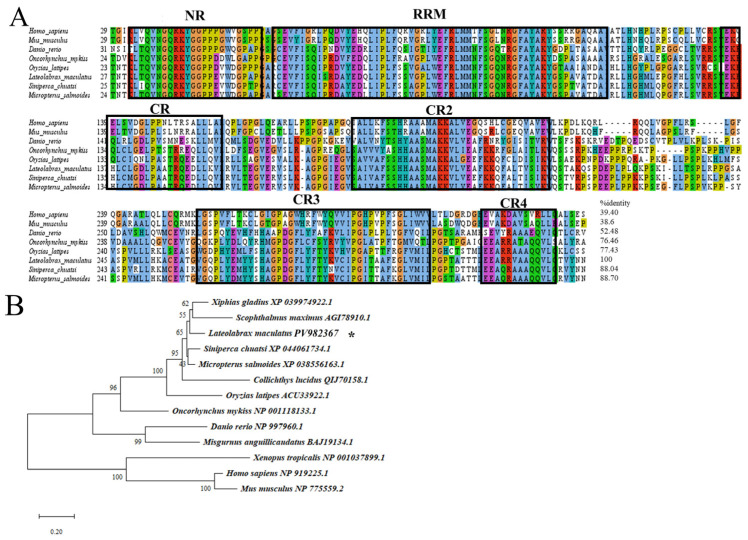
Multiple alignment and phylogenetic tree of dnd aa of spotted sea bass. (**A**) Six conserved domains or motifs are indicated in the black block, including RNA recognition motif (RRM), N-terminal region (NR), and four C-terminal regions (CR1–4), which are typically present in dnd protein. (**B**) Phylogenetic tree generated using the neighbor-joining method based on dnd. Other accession numbers are indicated. * represents the species that are studied.

Multiple sequence alignment showed that the aa sequence of Lmdnd contained an RNA recognition motif (RRM) and five conserved regions, namely the N-terminal NR domain and the C-terminal CR1–4 domains (Figure 2A). Blast alignment showed that Lmdnd shared 61.46–88.70% identity with fish dnd sequences and 39.40–52.48% with other species (Figure 2A).

A phylogenetic tree was constructed based on several representative and well-studied aa sequences of teleosts and mammals Lmvasa and Lmdnd. The results showed that the vasa and dnd of teleosts were different from mammals. Further subdivision of teleosts suggests that Lmvasa was phylogenetically close to that of Japanese bass (*Lateolabrax japonicus*) (Figure 1B). In addition, Lmdnd was clustered with swordfish (*Xiphias gladius*) and turbot dnd proteins (Figure 2B).

### 3.2. Expression and Purification of Recombinant Lmvasa Protein

The recombinant protein His-Lmvasa was mainly expressed in inclusion bodies. The protein was purified using Ni^2+^-TED affinity chromatography and detected using SDS-PAGE analysis. Thick bands were observed at 75 kDa (Figure S3A,B). Anti-Lmvasa specific antibody against purified fusion protein was successfully generated in rabbits.

### 3.3. Lmvasa and Lmdnd Are Specifically Expressed in Germ Cells and During Embryonic Development

**Figure 3:**
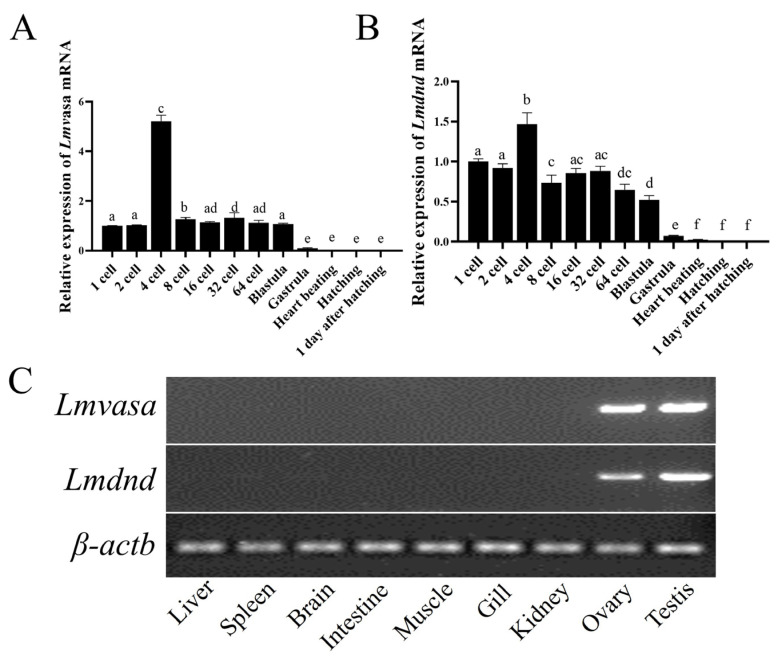
Spatial and temporal expression analyses of *Lmvasa* and *Lmdnd* isoforms detected by RT-PCR. (**A**) RT-qPCR of Lmvasa genes in different development stages of embryos. (**B**) RT-qPCR of Lmdnd genes in different development stages of embryos. *β-actin* as the control. (**C**) Values with the different superscript letters are significantly different (ANOVA, *p* < 0.05). Tissue specific expression of and *Lmvasa* and *Lmdnd* determined by RT-PCR. *β-actin* served as reference. M, DNA size marker.

RT-qPCR and RT-PCR were conducted to explore the expression of *Lmvasa* and *Lmdnd* during embryonic development and in different tissues. RT-qPCR analysis revealed that the expression of *Lmvasa* and *Lmdnd* were detected in the early stages of development, from embryos until the blastula stage. *Lmvasa* mRNA expression began at the 1–2-cell stage, increased significantly at 4-cells, then decreased gradually, and remained stable from the 8-cell stage to the blastocyst stage. After the gastrula stage, the expression of *Lmvasa* decreased significantly, and eventually became undetectable (Figure 3A). Similarly, *Lmdnd* mRNA reached its highest expression level at the 4-cell stage, followed by a gradual decrease in expression, and a significant decrease after the gastrula stage until it became undetectable (Figure 3B). Analysis by RT-PCR in different tissues showed that *Lmvasa* and *Lmdnd* were detected almost exclusively in the ovary and testis, as almost no expression was detected in other tissues (Figure 3C).

**Figure 4:**
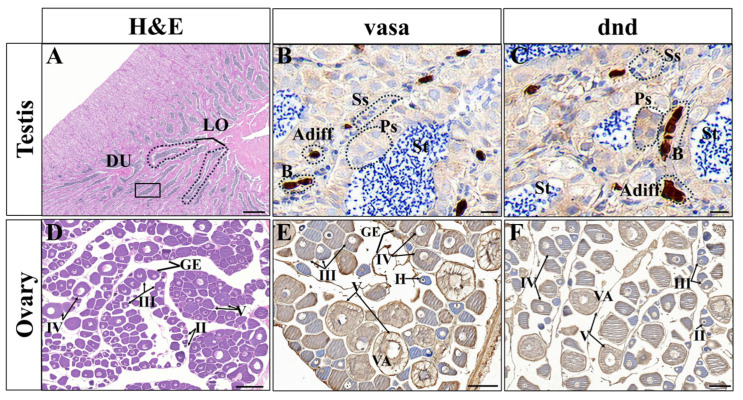
Expression of spotted sea bass Lmvasa and Lmdnd proteins in the ovary and testis. (**A**,**D**) Histological sections of testis and ovary stained with Hematoxylin and Eosin (H&E). (**B**,**C**) Immunohistochemical sections of testis incubated with anti-Lmvasa and anti-dnd1 antibodies. Lmvasa and Lmdnd proteins were most intense in spermatogonia, faint in spermatocytes, and had disappeared in spermatids. (**E**,**F**) Immunohistochemical sections of ovary incubated with anti-Lmvasa and anti-dnd1 antibodies. In ovary, Lmvasa and Lmdnd were granular around the nucleus in stage II oocytes and weakly expressed in stage III oocytes. They showed weak diffusion in cytoplasm in stage IV oocytes and were evenly distributed in follicles in stage V oocytes. DU, sperm duct; LO, lobular lumen; Adiff, Type A differentiated spermatogonia; B, Type B spermatogonia; Ps, primary spermatocytes; Ss, secondary spermatocytes; St, Spermatids; GE, germinal epithelium; VA, vascuoles; II, primary growth oocyte; III, perinucleolar or previtellogenic oocyte; IV, late vitellogenic oocyte; and V, postvitellogenic oocyte, described by Yang et al. [44]. Scale bars: 200 μm (**A**,**D**), 10 μm (**B**,**C**), 100 μm (**E**,**F**).

IHC was carried out to investigate the localization of Lmvasa and Lmdnd proteins in the gonads. In the finger-like seminiferous lobules of the testis, different types of germ cell sacs and single spermatogonia were distributed in the germinal epithelium (Figure 4A). The signals for Lmvasa and Lmdnd proteins were most intense in spermatogonia, faint in spermatocytes, and absent in spermatids (Figure 4B,C). In the mature female ovary, Lmvasa and Lmdnd proteins were expressed at all stages of ovarian development, but expression was mainly concentrated in late vitellogenic oocytes (LVO, IV) and postvitellogenic oocytes (V). Expression was weak in primary growth oocytes (II) and perinucleolar (PNO) and previtellogenic oocytes (III) (Figure 4E,F). In stage II oocytes, Lmvasa and Lmdnd were expressed in granules around the nucleus, and they were weakly expressed in stage III oocytes. Lmvasa and Lmdnd were diffused weakly in the cytoplasm in stage IV oocytes. In stage V oocytes, Lmvasa and Lmdnd proteins were evenly distributed in the follicles.

### 3.4. Vasa and Dnd RNA Localization in Spotted Sea Bass Visualized by WISH

**Figure 5:**
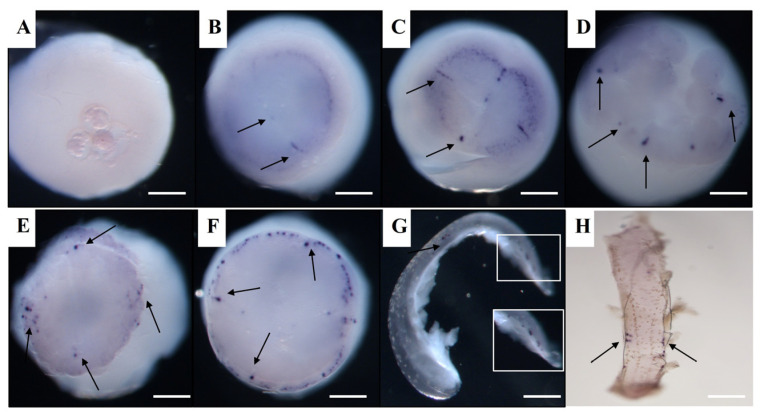
Localization of *Lmvasa* mRNA during embryonic development based on WISH. (**A**) 1-cell; (**B**) 2-cell; (**C**) 4-cell; (**D**) 16-cell; (**E**) gastrula; (**F**) early gastrula; (**G**) late gastrula; and (**H**) somite stages. Black arrows indicate regions of *Lmvasa* transcript aggregation (dark purple). Magnification of the boxed area in (**G**). Scale bar: 200 μm. WISH, whole mount in situ hybridization.

**Figure 6:**
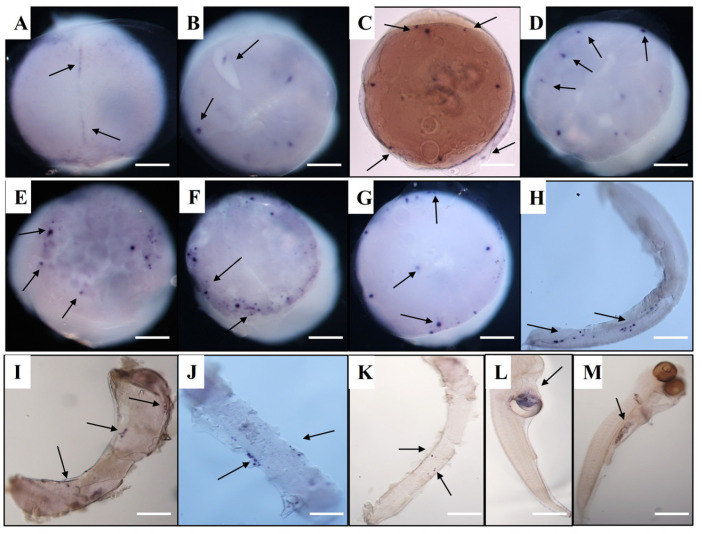
Localization of *Lmdnd* mRNA during embryonic development based on WISH. (**A**) 2-cell; (**B**) 4-cell; (**C**) 8-cell; (**D**) 32-cell; (**E**) morula; (**F**), blastula; (**G**) early gastrula; (**H**) late gastrula; (**I**) early somite; **(J**) late somite; (**K**) heart beating; and (**L**) hatching stages; (**M**) 3 days after hatching. Black arrows indicate *Lmdnd* transcript aggregated regions (dark purple). Scale bar: 200 μm. WISH, whole mount in situ hybridization.

At the cleavage stages, *Lmvasa* and *Lmdnd* mRNA aggregated along the cleavage planes before being localized within several individual cells at the morula stage. At the 1-cell stage, *Lmvasa* and *Lmdnd* mRNA could not be detected using WISH (Figure 5A). At the 2-cell stage, many small signals of *Lmvasa* and *Lmdnd* mRNA were detected within the forming cleavage furrow and around the cell margin of the first cleavage furrow (Figure 5B and Figure 6A). At the 4-cell stage, four *Lmvasa* and *Lmdnd* signals were found in the first and second cleavage furrow, and each of the signals remained localized at the outer edge of the cleavage plane (Figure 5C and Figure 6B). At the 8-cell stage, four new *Lmdnd* signals were present along the two new cleavage furrows (Figure 6C), and at the 16–32 cell stage, eight *Lmvasa* and *Lmdnd* signals were still localized in the cleavage furrow (Figure 5D and Figure 6D).

At the morula stage, *Lmdnd* signals were observed within individual cells. The signals were usually in four to six large clusters within individual cells, with smaller fragments in neighboring cells (Figure 6E). At the blastula stage, more *Lmvasa* and *Lmdnd* signal-expressing cells were observed in each group, and most of the positive cells were distributed at the edge of the blastoderm to form PGCs (Figure 5E and Figure 6F). At the early gastrula stage, *Lmvasa*-and *Lmdnd*-positive cells were predominantly located at the edge of the blastoderm, but a few signals were occasionally observed at the upper or middle part of the blastoderm (Figure 5F and Figure 6G). During the late gastrula stages, *Lmvasa*-and *Lmdnd*-positive cells moved towards the posterior part of the embryo as the embryonic body was extended (Figure 5G and Figure 6H).

**Figure 7:**
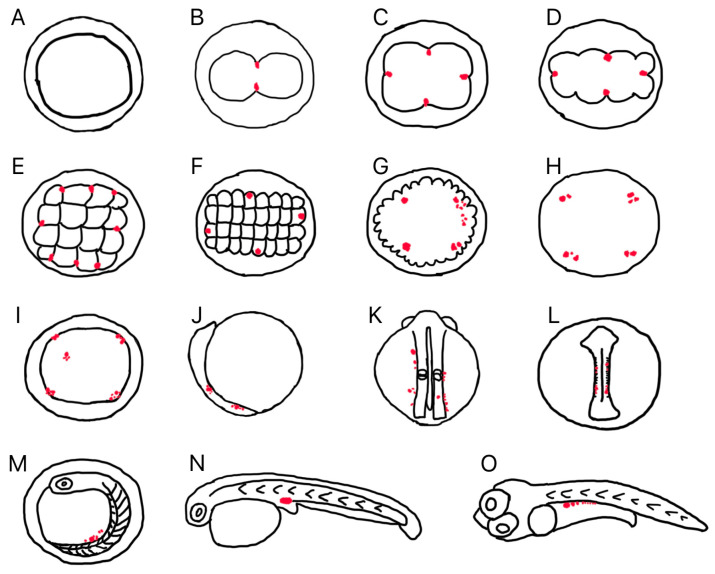
Schematic of *Lmvasa* or *Lmdnd* signal distribution during embryogenesis in spotted sea bass. Red spots indicate *Lmvasa* or *Lmdnd* signals. (**A**) 1-cell; (**B**) 2-cell; (**C**) 4-cell; (**D**) 8-cell; (**E**)16-cell; (**F**) 32-cell; (**G**) morula; (**H**) blastula; (**I**) early gastrula; (**J**) late gastrula; (**K**) early somite; (**L**) late somite stage; (**M**) heart beating; and (**N**) hatching stages. (**O**) 3 days after hatching.

As the embryo developed further, *Lmdnd*-positive cells migrated towards the embryonic body in the early somite stage and were loosely arranged on both sides of the embryonic body from the anterior side of the embryonic axis (head region) to the caudal bud (Figure 5H and Figure 6I). At the late somite stage, the bilateral clusters formed by *Lmvasa*-and *Lmdnd*-positive cells actively migrated posteriorly (Figure 6J) and gathered into two clusters at the intended genital crest site during the heart beating stage (Figure 6K). At the hatching stage, *Lmdnd*-positive cells were located below the spine at the junction of the yolk bulb and yolk sac extension (Figure 6L). Finally, *Lmdnd*-positive cells were located at the dorsal side of the gut and the ventral side of the notochord at 3 days after hatching (dah) (Figure 6M). Figure 7 is a schematic illustration of the spatiotemporal expression pattern of *Lmvasa* and *Lmdnd* during spotted sea bass embryogenesis.

### 3.5. Visualization of Three Fish PGCs by GFP-Lmvasa 3′-UTR mRNA

**Figure 8:**
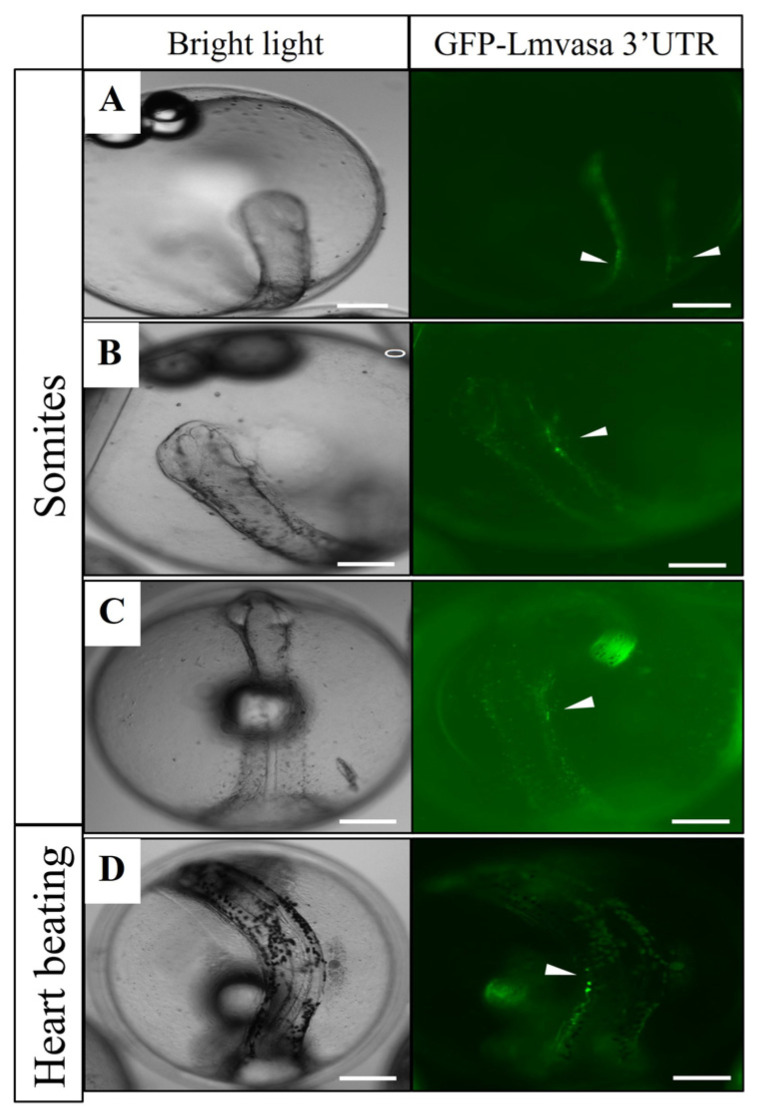
Migration of labeled PGCs in spotted sea bass embryo. GFP-*Lmvasa* 3′UTR mRNA was injected into the vegetal pole of 1-to 4-cell stage embryos. (**A**–**C**) Somite stage; (**D**) heart-beating stage. White arrows indicate GFP-positive cells (PGCs). Concentrations of injected mRNA were all 300 ng/μL. Scale bar: 200 μm.

At the somite stage, weak GFP-positive cells (PGCs) were observed on one or both sides of the embryonic body (Figure 8A–C). At the heart beating stage, PGCs migrated dorsally and were closer to the gonadal ridge (Figure 8D). These results suggest that GFP-*Lmvasa* 3′-UTR mRNA can label the PGCs of spotted sea bass.

**Figure 9:**
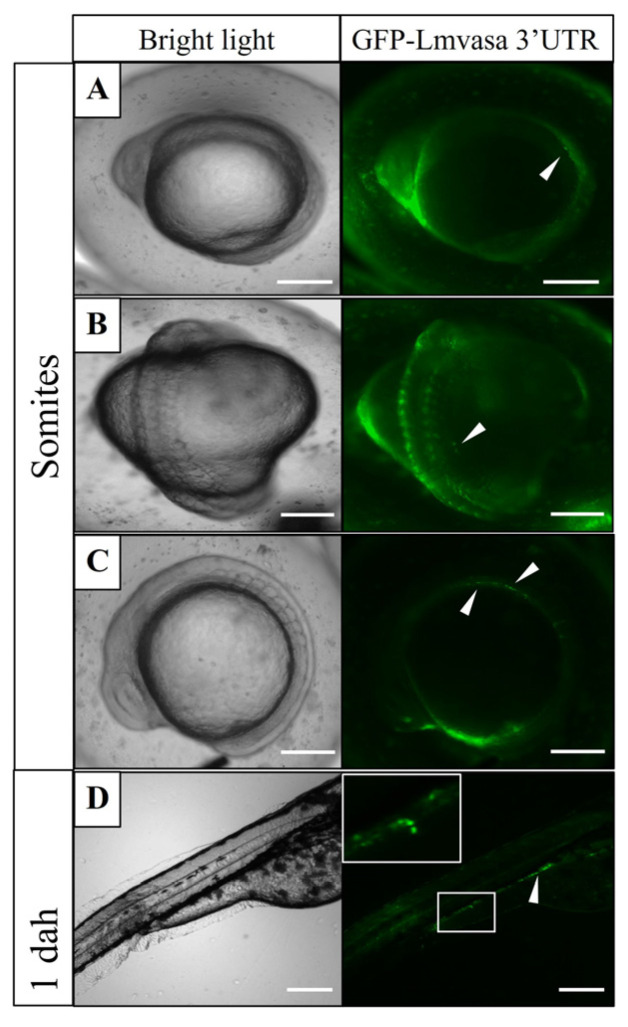
Visualization of zebrafish PGCs following injection of GFP-*Lmvasa* 3′-UTR mRNA. (**A**–**C**) Somite stage; (**D**) 1 day after hatching (1 dah). Magnification of the boxed areas in (**D**) showing localization of GFP-*Lmvasa* 3′UTR signals in PGCs. White arrows indicate GFP-positive cells (PGCs). Scale bar: 200 μm.

**Figure 10:**
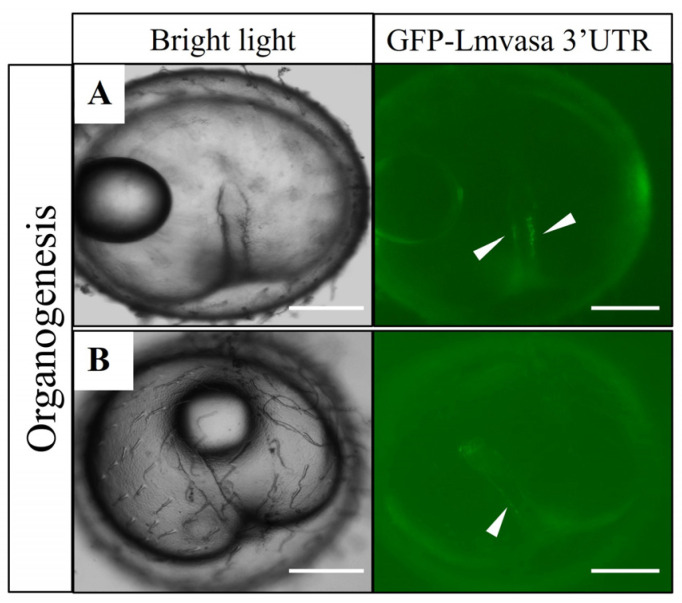
Visualization of medaka PGCs following injection of GFP-*Lmvasa* 3′-UTR mRNA. (**A**,**B**) Organogenesis stage. White arrows indicate GFP-positive cells (PGCs). Scale bar: 200 μm.

To verify the functional conservation of the 3′-UTR sequence in spotted seabass, GFP-*Lmvasa* 3′-UTR mRNA was injected into the embryos of zebrafish and medaka. At the somite stage, PGCs with green fluorescence were observed in the embryonic body of zebrafish (Figure 9A,B). As the embryo developed further, PGCs moved towards the dorsal side of the embryo (Figure 9C). At 1 dah, PGCs aligned at the dorsal side of the peritoneal cavity where the gonad would eventually form (Figure 9D). At the organogenesis stage of medaka, PGCs with green fluorescence were localized in the embryos (Figure 10A,B). These results suggest that the GFP-*Lmvasa* 3′-UTR mRNA can label PGCs in two other types of fish: zebrafish (early localization) and medaka (loss of early specific localization).

## 4. Discussion

In this study, *Lmvasa* and *Lmdnd* were identified as both sex marker genes for spotted sea bass. The vasa protein of spotted sea bass, like those of other teleosts, has a conserved domain of the typical DEAD-box protein family, which is involved in ATPase-dependent RNA binding and unwinding activity [14,45,46,47]. The results further confirmed that the isolated vasa gene was a vasa homologous gene, and that vasa was evolutionarily conserved. The dnd protein has an RNA-binding protein with a typical RRM motif in spotted sea bass, which is consistent with those of zebrafish [48] and medaka [22].

Phylogenetic analysis showed that aa sequences of Lmvasa and Lmdnd were highly homologous to those of teleosts. RT-qPCR results showed that *Lmvasa* and *Lmdnd* were expressed from early embryonic development to blastula stage. Zebrafish [23], medaka [49], and *P. Mangurus* [29] has a similar expression trend in the embryonic stage. This expression trait occurs in the embryonic stage, possibly after the gastrulation stage, when somatic cells proliferate faster than germ cells [50]. These results confirm the high activity of these genes in early ontogeny, as they are crucial during the PGC formation stage.

*Lmvasa* and *Lmdnd* mRNA were only expressed in gonad tissue, which was also reported for medaka [51], summer flounder (*Paralichthys dentatus*) [52], and spinyhead croaker (*Collichthys lucidus*) [31]. This indicates that *Lmvasa* and *Lmdnd* homologs can serve as specific molecular markers for spotted sea bass germ cells. Studies have shown that dnd mRNA exhibits sexual dimorphism in adult gonads across species. For example, dnd expression was detected only in mouse testis [27], whereas in *Xenopus* [28] and *P. mangurus* [29], its expression was restricted to the ovary. Our results showed that *Lmdnd* mRNA was expressed in both gonads in spotted bass. IHC analysis showed that in the testis, the signal of Lmvasa and Lmdnd proteins was strongest in spermatogonia, weak in spermatocytes, and absent in spermatocytes. Lmvasa and Lmdnd proteins are expressed in the ovaries at various stages of oogenesis. These phenomena have also occurred in Chinese hooknose carp (*Opsariichthys bidens*) [53], rainbow trout [54], and medaka [55] and turbot [16]. These results suggest that vasa and dnd play an important role in germ cells development as germ cells marker genes in spotted sea bass. In addition, a specific Lmvsas antibody against spotted bass germ cells was generated in this study. This tool will facilitate the preparation and purification of spermatogonia or eggs, providing a new solution for germ cell transplantation or cryopreservation.

WISH and mRNA microinjection techniques were used to label and track PGCs during embryonic development. The migration patterns of PGCs are not exactly the same among different fish species [56]. In this study, it was found that PGC of spotted bass belonged to the early localization pattern. In both species, the early germ is located in the cleavage furrow, and PGCs form at the blastula stage, similar to zebrafish [18,57]. The difference is that during somite formation, spotted sea bass PGCs are loosely arranged on both sides of the embryonic body, and occasionally ectopic PGCs are found near the brain, which is similar to the patterns reported for turbot [52] and goldfish (*Carassius auratus*) [58]. In contrast, zebrafish PGCs aggregate in clusters on the outside of the embryo. These ectopic PGCs in the brain may be due to cell mixing at the blastula stage and convergence and extension movement in the gastrula stages.

In many animals, PGCs inherit a special type of cytoplasm, the germ plasm, which distinguishes these cells from somatic cells. *Vasa* RNA is a component of germ plasm and can be localized by a germ plasm localization signal within the *vasa* 3′-UTR in different teleosts [18]. The zebrafish *vasa* 3′-UTR with early localization contains a rich set of positioning elements that can be used to label a wide variety of fish PGCs. For example, the PGCs of trout can be visualized using the *vasa* 3′-UTR from zebrafish [59], and the PGCs of half-smooth tongue sole (*Cynoglossus semilaevis*) can be visualized using the *vasa* 3′-UTR from medaka [60]. However, not all fish PGCs can be labeled with chimeric mRNA containing the *vasa* 3′-UTR from other fish. For example, GFP-*vasa* 3′-UTR mRNA from medaka and red seabream (*Pagrus major*) failed to label zebrafish PGCs [30]. In this study, the synthesized *Lmvasa* 3′-UTR mRNA was injected into embryos of multiple spotted sea bass, zebrafish, and medaka, respectively. It was found that PGCs could be labeled in all three fish, although the evolutionary relationship between spotted sea bass and zebrafish was very distant. Zhou et al. [61] reported that *vasa* 3′-UTR in black rockfish (*Sebastes schlegelii*) can label zebrafish and medaka PGCs because the black rockfish *vasa* 3′-UTR contains elements common to other fish, as well as the same three functional elements as zebrafish *vasa*. Thus, the *vasa* 3′-UTR of different teleosts differs in the labeling of zebrafish PGCs, which may be due to specific functional elements in the 3′-UTR. These differences also indicate that the mechanism of *vasa* 3′-UTR mRNA localization in teleost is complex and requires further investigation.

## 5. Conclusions

In our study, we validated *Lmvasa* and *Lmdnd* as key PGC-specific marker genes and identified the migration of these marker genes in early embryos of spotted bass, revealing the origin of PGC and its early distribution patterns.

## Figures and Tables

**Table 1:** Sequences of primers used in the present study ^a^.

Primer Name	Sequence 5′-3′	Size (bp)	Temperature, °C	Purpose
*vasa* oF	ATGGACGAATGGGAAGAAGAAGGAAC	1905	56	ORF
*vasa* oR	CTACTCCCATTCTTCATCATCAGCTG			
*dnd* oF	ATGGAGATGATGGAGAACAAGCGGAGC	1158	56	
*dnd* oR	TCAGTGGGCAAACTGGTTATTGTACACC			
5′RACE-*vasa*	TTCCGATTCTCGTCACCACC	292	70	RACE
3′RACE-*vasa*	TATTGGAAGAACTGGCCGCT	619	68	
5′RACE-*dnd*	CAGCTCCTGAACACGCTCAAGGT	300	70	
3′RACE-*dnd*	GGATAACGGCCGCCTTTGAAGGGC	414	68	
*vasa* qF	GTGGAACACCAGGGAGACTG	169	60	RT-qPCR
*vasa* qR	TGACGGTTCTCTTTGGACGG			
*dnd* qF	GCACGGAGAAGAGACACCTC	145	60	
*dnd* qR	ATGGCAGACACCCCCTCTAT			
*vasa* F	CCCACTATGAGACGGGCATC	394	56	RT-PCR
*vasa* R	CCAAAGGCAAACTTCCTGGC			
*dnd* F	ACCTTGAGCGTGTTCAGGAG	377	56	
*dnd* R	GAGGTGTCTCTTCTCCGTGC			
*β-actin F*	CAACTGGGATGACATGGAGAAG	114	58	
*β-actin R*	TTGGCTTTGGGGTTCAGG			
*vasa*-28a F	TGGGTCGCGGATCCGAATTCATGGACGA ATGGGAAGAAGAAG	1942	60	WISH
*vasa*-28a R	TGGTGGTGGTGGTGCTCGAGCTCCCATTCT TCATCATCAGCT			
WISH-*vasa* F	GCAGCTGACTTTCTCAAGACGGA	1013	56	
WISH-*vasa* R	AATTTTTCTTTTTTATTGGTGATC			
WISH-*dnd* F	ATGGAGATGATGGAGAACAAGCGGAGC	1158	56	
WISH-*dnd* R	TCAGTGGGCAAACTGGTTATTGTACACC			
3′UTR-*vasa* F	ATGAACTATACAAACTCGAGAAGGAATATTAGAGAAGC	327	56	3′UTR
3′UTR-*vasa* R	ATGAACTATACAAATAACTCGAGAAACCCATTAACCAATTTTTCT			

^a^ The underlined bases are linearized vector end sequences.

## Data Availability

The original contributions presented in this study are included in the article/Supplementary Materials. Further inquiries can be directed to the corresponding author.

## Supplementary Materials

The following supporting information can be downloaded at: https://www.mdpi.com/article/10.3390/genes16091012/s1, Figure S1: Full-length cDNA and aa sequences of Lmvasa from spotted sea bass; Figure S2: Full-length cDNA and aa sequences of Lmdnd from spotted sea bass; Figure S3: Expression of recombinant Lmvasa protein.

## Abbreviations

The following abbreviations are used in this manuscript:

PGCs	Primordial germ cells
dnd	Dead end
WISH	Whole mount in situ hybridization
ISH	In situ hybridization
3′-UTR	3′ untranslated region
GFP	Green fluorescent protein
H&E	Hematoxylin-eosin
HRP	Horseradish peroxidase
RRM	RNA recognition motif
dah	days after hatching
