# Supplementary material for: Genetic Characterization of Primordial Germ Cells in Spotted Sea Bass (Lateolabrax maculatus)
Source: Genes (Basel). 2025 Aug 27;16(9):1012. doi: 10.3390/genes16091012 (PMC12470244; doi:10.3390/genes16091012)
Supplement: Supplementary file 1 [file genes-16-01012-s001.zip › Supplementary Figures Legends.docx]

**Supplementary Figures Legends**

**Figure S1.** Full-length cDNA and amino acid sequences of Lmvasa from spotted sea bass. Start codon, stop codon, and putative polyadenylation signal are shown in bold. The yellow region represents RG repeats, the green region represents RGG repeats, and the red region represents the eight conserved motifs of the DEAD-box family described by Gustafson [45].

**Figure S2.** Full-length cDNA and amino acid sequences of Lmdnd from spotted sea bass. Start codon, stop codon and putative polyadenylation signal are shown in bold.

**Figure S3.** Expression of recombinant Lmvasa protein. (A) Recombinant expression of His-vasa in Escherichia coli. Lane M, protein molecular mass marker; lane 1, total proteins from E. coli without IPTG induction; lane 2, total proteins from E. coli with IPTG induction; lane 3, total protein isolated from precipitates (inclusion bodies) of E. coli; lane 4, total protein isolated from supernatant of E. coli. Ni^2+^-TED affinity chromatography was used to collect the flow (lane 5) and wash (lane 6) from inclusion bodies. Lane 7 and 8, purified His-vasa protein. (B) Recombinant vasa protein detected using western blotting with specific antibodies.
