# Supplementary figures and images for: Genetic Characterization of Primordial Germ Cells in Spotted Sea Bass (Lateolabrax maculatus)
Source: Genes (Basel). 2025 Aug 27;16(9):1012. doi: 10.3390/genes16091012 (PMC12470244; doi:10.3390/genes16091012)

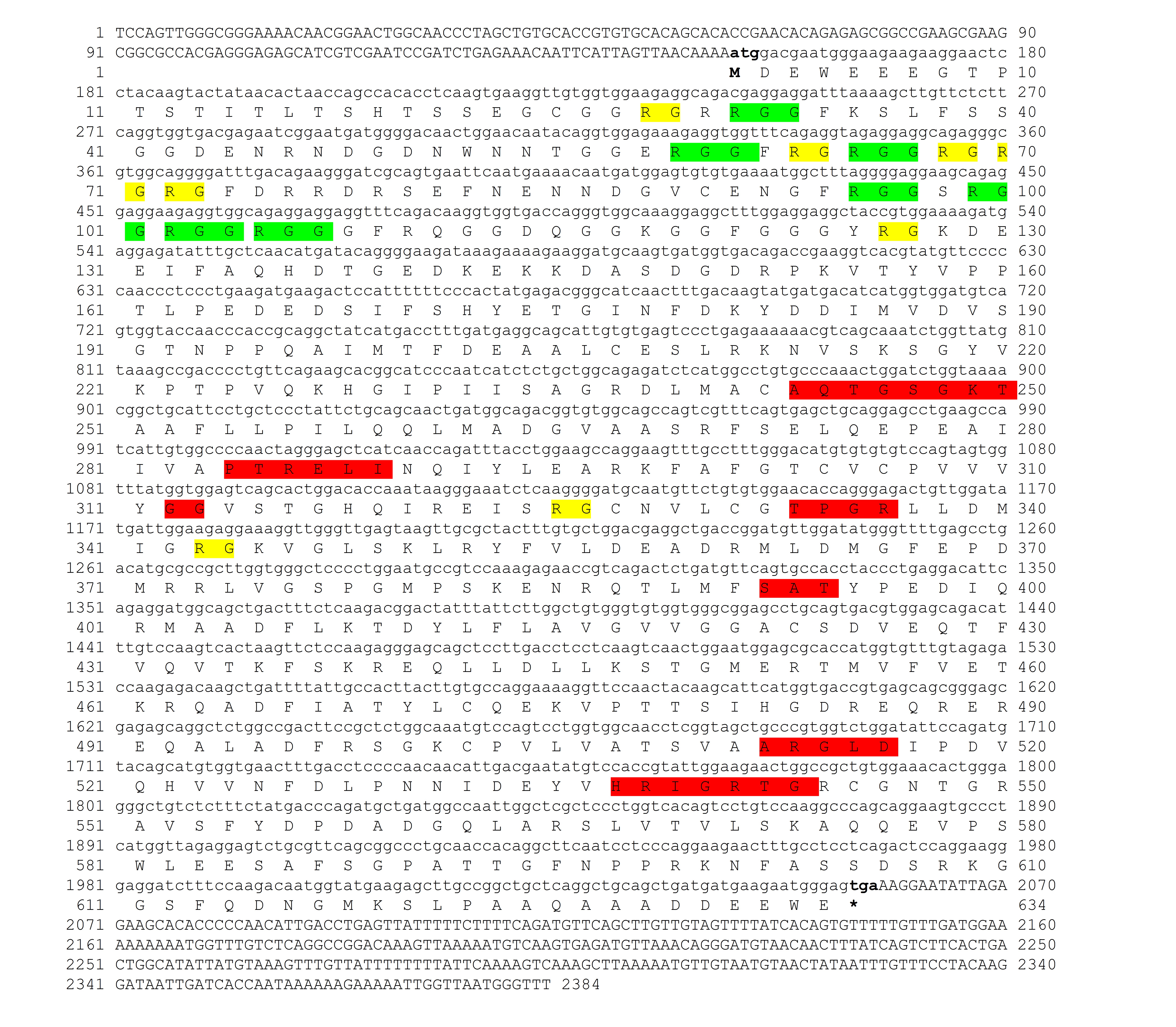

Supplement: Supplementary file 1 [file genes-16-01012-s001.zip › Figure S1.jpg]

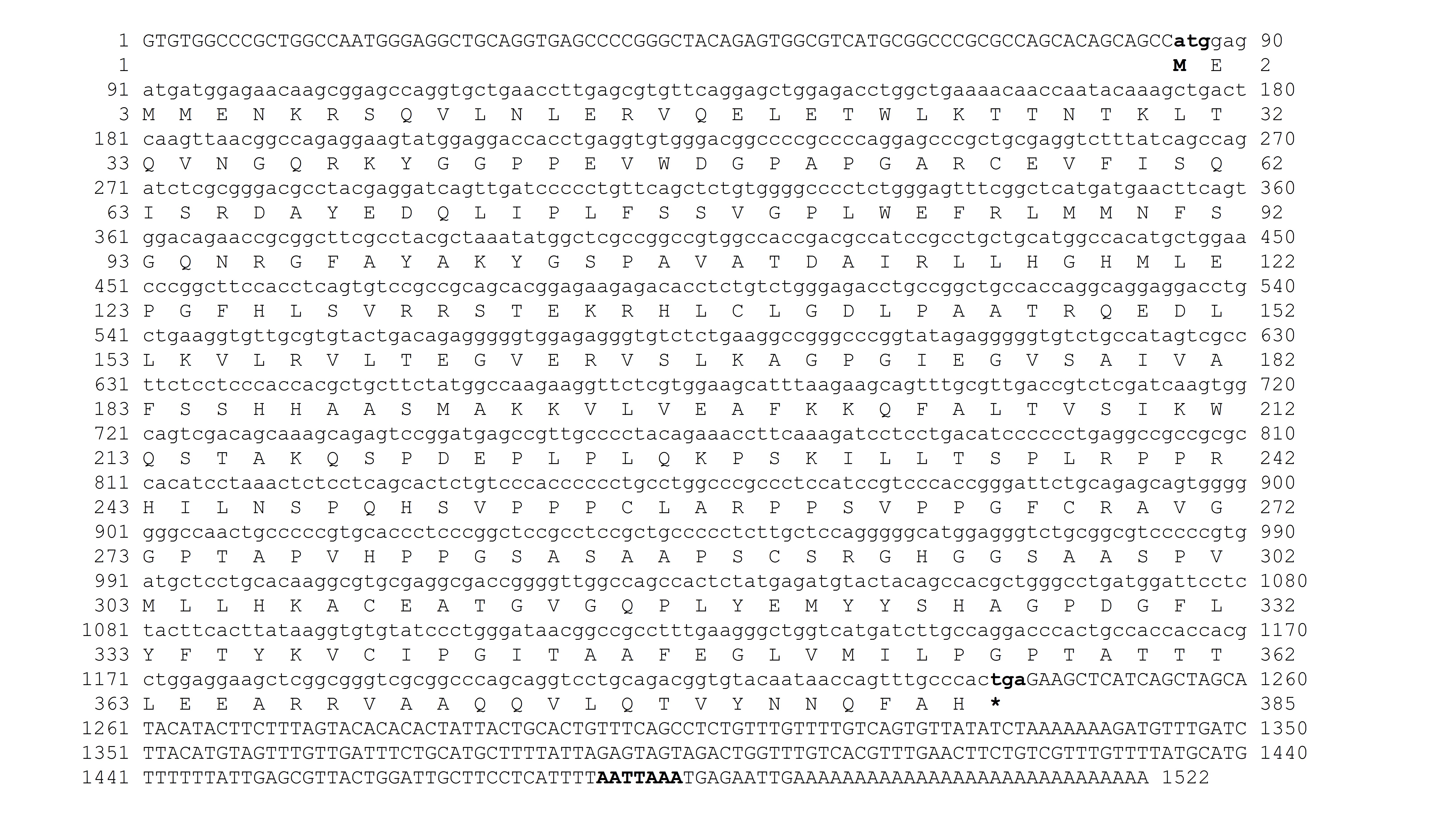

Supplement: Supplementary file 1 [file genes-16-01012-s001.zip › Figure S2.jpg]

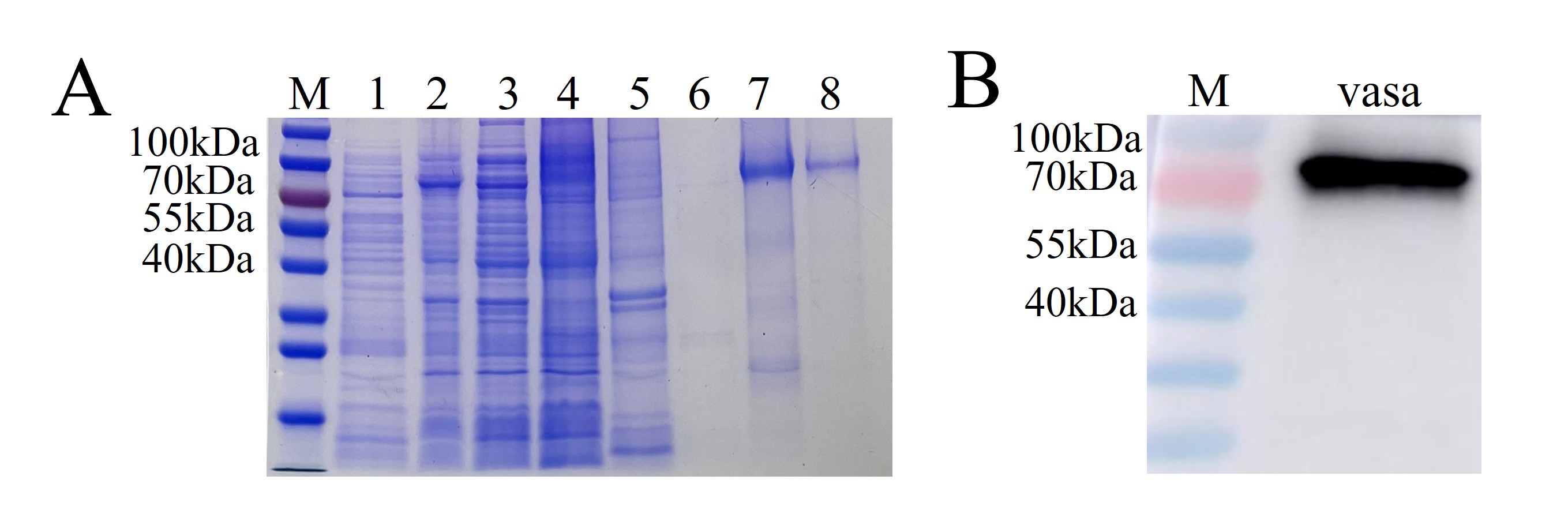

Supplement: Supplementary file 1 [file genes-16-01012-s001.zip › Figure S3.jpg]
